# Implementing a nurse-enabled, integrated, shared-care model involving specialists and general practitioners in breast cancer post-treatment follow-up: a study protocol for a phase II randomised controlled trial (the EMINENT trial)

**DOI:** 10.1186/s13063-020-04740-1

**Published:** 2020-10-15

**Authors:** Raymond Javan Chan, Jon Emery, Katharine Cuff, Laisa Teleni, Camilla Simonsen, Jane Turner, Monika Janda, Daniel Mckavanagh, Lee Jones, Emma McKinnell, Melissa Gosper, Juanita Ryan, Ria Joseph, Bethany Crowe, Jennifer Harvey, Marissa Ryan, Christine Carrington, Rebecca Nund, Megan Crichton, Steven McPhail

**Affiliations:** 1 Division of Cancer Services, Proncess Alexandra Hospital, Metro South Health, Brisbane, Queensland Australia; 2 School of Nursing, Queensland University of Technology, Brisbane, Queensland Australia; 3 Department of General Practice and Centre for Cancer Research, University of Melbourne, Melbourne, Victoria Australia; 4 Faculty of Medicine, University of Queensland & Royal Brisbane and Women’s Hospital, Brisbane, Queensland Australia; 5 Centre for Health Services Research, Faculty of Medicine, University of Queensland, Brisbane, Queensland Australia; 6 Institute of Health and Biomedical Innovation, Queensland University of Technology, Brisbane, Queensland Australia; 7 McGrath Foundation & Princess Alexandra Hospital, Metro South Health, Brisbane, Queensland Australia; 8 Division of Surgery, Princess Alexandra Hospital, Metro South Health, Brisbane, Queensland Australia; 9 Radiation Oncology Centre, Princess Alexandra Hospital, Metro South Health and University of Queensland, Brisbane, Queensland Australia; 10 Pharmacy Department, Princess Alexandra Hospital, Metro South Health, Brisbane, Queensland Australia; 11 School of Health and Rehabilitation Sciences, University of Queensland, Brisbane, Queensland Australia; 12 Australian Centre For Health Services Innovation (AusHSI), Queensland University of Technology, Brisbane, Queensland Australia; 13 Clinical Informatics Division, Metro South Health, Brisbane, Queensland Australia

**Keywords:** Early breast cancer, Oncology, Shared-care, Quality of life, Protocol, Randomised controlled trial

## Abstract

**Background:**

Due to advances in early detection and cancer treatment, 5-year relative survival rates for early breast cancer surpass 90% in developed nations. There is increasing focus on promotion of wellness in survivorship and active approaches to reducing morbidity related to treatment; however, current models of follow-up care are heavily reliant on hospital-based specialist-led care. This study aims to test the feasibility of the EMINENT intervention for implementing an integrated, shared-care model involving both cancer centre specialists and community-based general practitioners for early breast cancer post-treatment follow-up.

**Methods:**

We describe a protocol for a phase II, randomised controlled trial with two parallel arms and 1:1 allocation. A total of 60 patients with early-stage breast cancer will be randomised to usual, specialist-led, follow-up care (as determined by the treating surgeons, medical oncologists, and radiation oncologists) or shared follow-up care intervention (i.e. EMINENT). EMINENT is a nurse-enabled, pre-specified shared-care pathway with follow-up responsibilities divided between cancer centre specialists (i.e. surgeons and oncologists) and general practitioners. The primary outcome is health-related quality of life as measured by the Functional Assessment of Cancer Therapy—Breast Cancer. Secondary outcomes include patient experience, acceptance, and satisfaction of care; dietary, physical activity, and sedentary behaviours; financial toxicity; adherence; health resource utilisation; and adverse events.

**Discussion:**

The trial is designed to identify the barriers to implementing a shared-care model for breast cancer survivors following treatment. Results of this study will inform a definitive trial testing the effects of shared-care model on health-related quality of life of breast cancer survivors, as well as its ability to alleviate the growing demands on the healthcare system.

**Trial registration:**

Australia and New Zealand Clinical Trials Registry ACTRN12619001594112. Registered on 19 November 2019

## Administrative information

The order of the items has been modified to group similar items (see http://www.equator-network.org/reporting-guidelines/spirit-2013-statement-defining-standard-protocol-items-for-clinical-trials/).

**Table:** 

Title {1}	Implementing a nurse-enabled, integrated, shared-care model involving specialists and general practitioners in breast cancer post-treatment follow-up: a study protocol for a Phase II randomised controlled trial (The EMINENT Trial)
Trial registration {2a and 2b}.	Registry Name: Australia and New Zealand Clinical Trials Registry. Trial Identifier: ACTRN12619001594112
Protocol version {3}	24/03/2020, Version 2.0
Funding {4}	Metro South Health SERTA (Study, Education and Research Trust Account) grant.
Author details {5a}	Raymond Javan Chan, Division of Cancer Services, Princess Alexandra Hospital, Metro South Health & School of Nursing, Queensland University of Technology, Brisbane, Queensland, Australia. Email: raymond.chan@qut.edu.au Jon Emery, Department of General Practice and Centre for Cancer Research, University of Melbourne, Melbourne, Victoria, Australia. Email: jon.emery@unimelb.edu.au Katharine Cuff, Head of Haematology, Division of Cancer Services, Princess Alexandra Hospital, Metro South Health; Senior lecturer, University of Queensland; and Clinical Assoc Prof, Queensland University of Technology, Brisbane, Queensland, Australia. Email: Katharine.Cuff@health.qld.gov.au Laisa Teleni, Research Coordinator, School of Nursing, Queensland University of Technology, Brisbane, Queensland, Australia. Email: laisa.teleni@qut.edu.au Camilla Simonsen, Division of Cancer Services, Princess Alexandra Hospital, Metro South Health, Brisbane, Queensland, Australia. Email: Camilla.Simonsen@health.qld.gov.au Jane Turner, Faculty of Medicine, University of Queensland & Royal Brisbane and Women’s Hospital, Brisbane, Queensland, Australia. Email: jane.turner@uq.edu.au Monika Janda, Centre for Health Services Research, Faculty of Medicine, University of Queensland, Brisbane, Queensland, Australia. Email: m.janda@qut.edu.au Daniel Mckavanagh, Division of Cancer Services, Princess Alexandra Hospital, Metro South Health, Brisbane, Queensland, Australia. Email: Daniel.Mckavanagh@health.qld.gov.au Lee Jones, Institute of Health and Biomedical Innovation, Queensland University of Technology, Brisbane, Queensland, Australia. Email: lee.jones@qut.edu.au Emma McKinnell, Division of Cancer Services, Princess Alexandra Hospital, Metro South Health, Brisbane, Queensland, Australia. Email: Emma.McKinnell@health.qld.gov.au Melissa Gosper, McGrath Foundation & Princess Alexandra Hospital, Metro South Health, Brisbane, Queensland, Australia. Email: Melissa.Gosper@health.qld.gov.au Juanita Ryan, Division of Surgery, Princess Alexandra Hospital, Metro South Health, Brisbane, Queensland, Australia. Email: Juanita.Ryan@health.qld.gov.au Ria Joseph, School of Nursing, Queensland University of Technology, Brisbane, Queensland, Australia. Email: ria.joseph@qut.edu.au Bethany Crowe, Division of Cancer Services, Princess Alexandra Hospital, Metro South Health, Brisbane, Queensland, Australia. Email: bethany.crowe@health.qld.gov.au Jennifer Harvey, Radiation Oncology Centre, Princess Alexandra Hospital, Metro South Health and University of Queensland, Brisbane, Queensland, Australia. Email: Jennifer.Harvey@health.qld.gov.au Marissa Ryan, Pharmacy Department, Princess Alexandra Hospital, Metro South Health, Brisbane, Queensland, Australia. Email: Marissa.Ryan@health.qld.gov.au Christine Carrington, Department of Pharmacy, Princess Alexandra Hospital, Metro South Health & School of Pharmacy, University of Queensland, Brisbane, Queensland, Australia. Email: Christine.Carrington@health.qld.gov.au Rebecca Nund, School of Health and Rehabilitation Sciences, University of Queensland, Brisbane, Queensland, Australia. Email: r.nund@uq.edu.au Megan Crichton, School of Nursing, Queensland University of Technology, Brisbane, Queensland, Australia. Email: megan.crichton@qut.edu.au Steven McPhail, Australian Centre For Health Services Innovation (AusHSI), Queensland University of Technology, Brisbane, Queensland, Australia & Clinical Informatics Division, Metro South Health, Brisbane, Queensland, Australia. Email: steven.mcphail@qut.edu.au
Name and contact information for the trial sponsor {5b}	Professor Raymond Chan Division of Cancer Services, Princess Alexandra Hospital, Metro South Health, Brisbane, Queensland, Australia. And School of Nursing, Queensland University of Technology, Brisbane, Queensland, Australia. Email: Raymond.chan@qut.edu.au
Role of sponsor {5c}	The sponsor has absolute authority over securing funding, study design, data analysis, interpretation of data, writing of the report, decision to submit the report for publication.

## Introduction

### Background and rationale {6a}

In Australia, breast cancer is the most common cancer in females with an estimated 19,535 new cases annually [1]. With advances in early detection and cancer treatment, such as surgery, post-operative radiotherapy, and pre- or post-operative systemic therapies including cytotoxic chemo-, endocrine, and anti-HER2 antibody therapies, the 5-year relative survival rate for breast cancer is estimated at 91% [2, 3]. Consequently, in 2018, there were at least 200,000 breast cancer survivors living in Australia [4]. Despite good survival outcomes, breast cancer survivors require supportive care including prevention of cancer recurrence, surveillance for secondary or new primary cancer, and management of a range of long-term bio-psycho-social effects from their cancer diagnosis and treatment. In addition, many cancer survivors need management of comorbidities as they are 2.5 times more likely to develop mental and behavioural problems and almost 1.5 times more likely to develop musculoskeletal conditions, circulatory conditions, and endocrine system disorders compared with non-cancer patients [5]. These health concerns highlight the importance of a comprehensive, well-integrated, patient-centred model of care for people following completion of breast cancer treatment.

The current models of post-treatment care in Australia are mostly hospital-based and specialist-driven and focus on surveillance for disease recurrence, rather than the holistic care needs of cancer patients. This model of follow-up care limits the integration between specialist institutions and general practitioners (GPs), overloads the specialist system, and is unsustainable to meet the demands of the ever-growing population of cancer survivors. Specialist-based follow-up carries the burdens of travel and out-of-pocket costs such as those for parking. Those in non-metropolitan areas face more major disruptions to engage in specialist-based follow-up. Therefore, there is a strong case for an integrated, shared post-treatment follow-up care model for breast cancer survivors that involves both cancer specialists as well as care provided in the community by GPs. Such a shared-care model is consistent with Cancer Australia statements [6], the Optimal Care Pathway for Breast Cancer [7], and international guidelines [8]. In addition, the literature suggests that such a model is feasible, acceptable, safe, and more cost effective and patient-centred than current models used within Australia [9, 10].

Despite the promising evidence base, a shared follow-up care model in which specialists in the acute cancer care setting and GPs collaborate is not routinely implemented across Australia and many developed nations. Barriers to such shared care include the lack of a coordination between multiple providers, lack of patient and provider knowledge about the benefits of shared care and how to implement it, insufficient or delayed communication between cancer specialists and GPs, and lack of awareness of available support such as funding models, tools, and resources [11, 12]. These barriers could be overcome if a Specialist Cancer Nurse advises stakeholders (patient and GPs) of the benefits of shared-care, facilitates effective and timely care coordination, and acts as the conduit between the specialist cancer multidisciplinary team and the GPs at key transition time points, such as completion of definitive primary and adjuvant treatment [13].

### Objectives {7}

The objective of the study is to test the feasibility of a prospective, pragmatic randomised controlled trial (RCT) of the EMINENT intervention—a nurse-enabled, integrated, shared-care model involving cancer specialists and GPs for early breast cancer post-treatment follow-up.

### Trial design {8}

This phase II pilot RCT aims to assess the feasibility of a larger definitive clinical trial. Outcome data will be collected at four timepoints (or five if Booster Nurse Clinic is attended): (*t*_1_) baseline (at enrolment ± prior to Booster Nurse Clinic, if relevant), (*t*_2_) 3 months, (*t*_3_) 6 months, and (*t*_4_) 12 months.

## Methods: participants, interventions, and outcomes

### Study setting {9}

This study is conducted in a large, Australian metropolitan tertiary teaching hospital and general practices.

### Eligibility criteria {10}

The study population consists of patients with early-stage breast cancer (i.e. no-distant metastases) or ductal carcinoma in situ (DCIS). Patients will be eligible to participate from 2 weeks prior to completion of definitive treatment (surgery or adjuvant chemotherapy) and up to 18 months after completion of treatment. Patients meeting all of the following criteria are eligible for inclusion: diagnosis of curable, early breast cancer; receiving care at the Princess Alexandra Hospital; able to speak and read English; 18 years of age or older; ambulatory at the time of recruitment; Eastern Cooperative Oncology Group (ECOG) performance status 0 or 1; able to nominate a GP or GP clinic to be involved in their follow-up; and access to a telephone. Patients meeting any of the following criteria are excluded: presence of severe mental, cognitive, or physical conditions that would limit the patient’s ability to provide informed consent.

### Who will take informed consent? {26a}

Potential participants are identified by the research nurse or treating clinician during multidisciplinary team meetings. Participants are approached by their treating clinicians to gauge their interest in the study and gain verbal consent to being approached by the research team. Participants are then contacted by the research nurse, screened for eligibility, and provided with study information, and after a time of reflection (at least 24 h), they sign the consent form with the research nurse. Table 3 outlines the different phases of the study and data collection.

### Additional consent provisions for collection and use of participant data and biological specimens {26b}

Consent to access Medicare and Pharmaceutical Benefits Scheme (PBS) data on service use that qualifies under the Medicare Benefits Schedule (MBS) will be obtained, including relevant claims details (date of service, Medicare item number, and description) and costs details.

## Interventions

### Explanation for the choice of comparators {6b}

Survivorship care of breast cancer survivors following completion of treatment is an important issue, especially in light of improving survival rates [2]. The shared-care model between specialists and GPs focusses on the complex care needs of breast cancer survivors, rather than solely on disease recurrence, and may influence patient health outcomes and service outcomes [10].

### Intervention description {11a}

#### Arm 1

The control group will receive usual follow-up care supplemented with a survivorship booklet on *Living Well After Cancer* published by Cancer Council Australia [14]. The usual care follow-up arrangement is a specialist-led model as determined by the treating surgeon, medical oncologist, and radiation oncologist. This specialist-led follow-up care is not standardised and with follow-up activities and schedules depending on individual patient needs and the discretion of the treating clinicians.

#### Arm 2

EMINENT is a multi-faceted intervention that includes a pre-specified shared-care pathway for post-treatment follow-up. The design of the EMINENT intervention is informed by a number of Cancer Australia statements, the Optimal Care Pathway [15], the self-efficacy model [16, 17], the Capabilities for Supporting Prevention and Chronic Condition Self-Management framework [18], and our extensive pilot work including a systematic review [19] and observational studies [20–23]. Table 1 outlines the active ingredients of the EMINENT intervention.

**Table 1:** Active ingredients of the EMINENT model of care intervention for patients allocated to receive the EMINENT intervention

Active ingredient	Personnel involved	Specific activities
**Nurse Clinic** (30–60 min) *Mode: face to face or telehealth*	Specialist Cancer Nurse^a^ and patient	• Treatment summary • Survivorship care planning • Collaborative planning for health goals • Post-treatment education
**Booster Nurse Clinic**^**b**^ (10–30 min) *Mode: face to face or telehealth*	Specialist Cancer Nurse^a^ and patient	• Survivorship care planning • Collaborative planning for health goals
**Pharmacist Consultation**^**c**^ (20 min) *Mode: face to face or telehealth*	Cancer Pharmacist and patient	• Medication reconciliation • Medication education
**Case conference with GP** (5–30 min) *Mode: teleconference*	Specialist Cancer Nurse^a^ and GP (± one more healthcare team member)	• Nurse presents treatment summary and survivorship care plan • Follow-up responsibilities of the GP negotiated • GP questions answered • Additional education and support provided to other healthcare team members (e.g. Practice Nurse)
**Shared follow-up care** *Mode: face to face or telehealth*	Cancer specialist, GP, and Specialist Cancer Nurse^a^ and patient	• Cancer specialist reviews patient, orders mammogram, and completes full physical examination every 6 months for 2 years post-diagnosis, then every 12 months up to 5 years post-diagnosis • GP reviews patient as per survivorship care plan at least every 12 months (e.g. for general health and comorbidity management, chronic disease management planning, psychosocial screening, management of cancer treatment toxicities and cancer-related symptoms, allied health referrals) • GP contacts nurse interventionist with any patient concerns

^a^Medical Oncology Clinical Nurse Consultant, Breast Care Nurse, or McGrath Breast Care Nurse

^b^Offered to patients who have delays in GP involvement greater than 3 months (up to 18 months)

^c^Offered to patients who have completed chemotherapy and/or are scheduled to receive aromatase inhibitor, or have completed surgery and radiotherapy

After enrolment, participants who have completed chemotherapy and radiotherapy or those who will receive aromatase inhibitor will participate in a 20-min telehealth cancer pharmacist consultation for medication reconciliation and education prior to Specialist Nurse consultation.

A 30–60-min consultation with a Specialist Cancer Nurse is then conducted to provide a treatment summary, the shared follow-up care appointment schedule, and survivorship patient education (including the survivorship booklet on *Living Well After Cancer* published by Cancer Council Australia [14]) and to co-develop a draft Survivorship Care Plan (SCP). The SCP includes up to three SMART (Specific, Measurable, Achievable, Realistic, and Timely) goals that are developed by the nurse and patient in partnership using motivational interviewing and self-efficacy techniques. Due to the recent COVID-19 pandemic, where there are delays of 3–18 months before GP involvement, a second ‘booster’ Specialist Cancer Nurse consultation is offered to patients to update the SCP. The treatment summary and draft SCP is provided to the GP.

Within 4 weeks of the Specialist Cancer Nurse consultation, a 5–30-min case conference between the Specialist Cancer Nurse and the patient’s nominated GP is completed to communicate the treatment summary and shared follow-up care schedule and to finalise the SCP and negotiate the GP’s role in facilitating the SCP goals. The GP may propose changes or express if they are not willing to take part in specific care activities outlined in the SCP. The finalised SCP is then filed in the patient’s medical records and provided to the patient and the GP.

The shared, follow-up care schedule consists of 6-monthly patient appointments with a cancer centre specialist and annual appointments with the GP for 2 years post-diagnosis. Following this, the schedule consists of alternating 6 monthly appointments with a cancer centre specialist and GP for up to 5 years post-diagnosis. At 5 years post-diagnosis, patients are discharged to the care of the GP, as per usual care. The GP appointments will focus on reviewing the SCP; promoting general health; primary prevention, screening, and management of comorbidities; psychosocial health; cancer treatment toxicities; cancer-related symptoms; chronic disease management planning; and allied health referrals. The GP has direct telephone access to the Specialist Cancer Nurse in case of concerns or escalation for acute care review. The cancer centre specialist appointments focus on surveillance activities such as physical examination and imaging (i.e. annual mammogram).

### Criteria for discontinuing or modifying allocated interventions {11b}

The presence of any of the following criteria constitutes cause for the withdrawal of the participant: altered mental capacity resulting in inability to provide continuing informed consent, notification from treating oncologist and/or GP that the participant is not deemed to have the capacity to consent, and recurrence or progressive disease or death.

### Strategies to improve adherence to interventions {11c}

Fidelity of the intervention will be assessed using the framework for behavioural interventions recommended by the National Institute of Health (NIH) [24, 25] as outlined in Table 2.

**Table 2:** Intervention fidelity strategies (adapted)

Goal	Strategies
Training providers	Specialist Cancer Nurses will be trained to standardise the delivery of the intervention to study participants. Training includes provision of study manual containing • Generic study information: standard operating procedures, study overview, reporting and documentation guidelines, communication flowchart, rationale for the study treatment, completion of survivorship care plan, self-management goal setting, and health coaching • Specialist Cancer Nurse-specific information: job description, intervention protocol, quality assurance, and monitoring An 8-h training program will be delivered by Experts in Cancer Survivorship and motivational interviewing. The program includes the National Cancer Nursing Education (EdCAN) learning module on survivorship, related literature, didactic presentations, and roleplay covering: basic concepts of quality cancer survivorship care, components of a high-quality treatment summary and survivorship care plan; provision of self-management support (including collaborative goal setting; motivational interviewing); and MBS item numbers that facilitate the proposed Model of Care.
Delivery of intervention	Intervention procedures are monitored through completion of intervention component checklists to ensure that the intervention is delivered as intended. Intervention checklists are completed during Clinics and GP Case Conferences to track protocol deviations across Specialist Cancer Nurses and study Arms. The intervention fidelity is closely monitored and discussed during the weekly 30-min meeting for the first 3 months of the trial between the Specialist cancer Nurses, research nurses, and investigators. Minimising contamination between conditions by training interventionists to address participant questions about randomisation and their assigned condition using non-biased explanations.
Receipt of intervention	The SCP serves as a resource for a participant to understand and refer to whenever they are unsure of follow-up schedule and collaborative goal setting.
Enactment of treatment skills	Enactment of treatment skills includes processes to monitor and improve participant ability to perform treatment-related behavioural skills and cognitive strategies in relevant real-life settings as intended. This goal will be achieved by ensuring participants are aware of the follow-up schedules and responsibilities of all health professionals, ensuring participants will have a copy of the completed SCP including all care responsibilities and goals set for the individual, and checking in with participants once in the first week into the model, then monthly/bimonthly until the end of the trial period as resources allow.

### Relevant concomitant care permitted or prohibited during the trial {11d}

No concomitant care or intervention is prohibited during the trial.

### Provisions for post-trial care {30}

There is no ancillary or post-trial care for participants in this trial. However, it is expected that the SCP generated will have the value of informing longer term updates of the SCP and future survivorship care.

### Outcomes {12}

The feasibility outcomes are recruitment and acceptability of the intervention. The primary endpoint is health-related quality of life (HRQoL) as measured by Functional Assessment of Cancer Therapy—Breast Cancer (FACT-B) [26] at baseline, 3, 6, and 12 months post-enrollment. The 37-item FACT-B is a valid and reliable tool for use in cancer survivors undergoing as well as beyond treatment and has been demonstrated to be sensitive to changes over time [27]. A total score as well as scores for each of the five subscales (physical, social/family, emotional, functional wellbeing, additional breast cancer concerns) are calculated, where higher scores indicate higher quality of life. FACT-B captures key domains of HRQoL and key symptoms that are relevant to the study population and sensitive to the EMINENT intervention.

Additional outcomes include a range of patient-reported secondary endpoints, and process outcomes related to implementation as shown in Table 3. Participants of the intervention group, as well as their nominated carer, breast cancer nurses, GPs, and other healthcare providers including other nurses, and hospital- and community-based rehabilitation providers will be invited to participate in a semi-structured interview. Open ended questions (Online Supplementary Material 1) will explore key factors that facilitate or hinder the implementation of the EMINENT intervention.

**Table 3:** Schedule for data collection during the EMINENT trial

	Study period						
	Enrolment	Allocation	Post-allocation				Close-out
Timepoint	***-t***_**1**_	***0***	***t***_**1**_	***t***_**2**_	***t***_**3**_	***t***_**4**_	***t***_***x***_
**Enrolment**							
Eligibility screen	X						
Informed consent	X						
Allocation		X					
**Interventions**							
EMINENT							
Usual Care							
**Primary endpoints**							
Health-related quality of life			**X**	**X**	**X**	**X**	
**Secondary endpoint**							
Patient experience of care			**X**				
Dietary behaviours			**X**	**X**	**X**	**X**	
Physical activity and sedentary behaviours			**X**	**X**	**X**	**X**	
Financial toxicity			**X**		**X**	**X**	
Adherence to clinical assessments							**X**
Health resource utilisation				**X**	**X**	**X**	**X**
Satisfaction of care						**X**	
Acceptance of intervention						**X**	
**Participant characteristics**							
Demographics			**X**				
Clinical characteristics			**X**				
**Process outcomes**							
Completion of intervention component						**X**	
Clinical encounters at cancer centre							**X**
Referrals back to acute care							**X**
GP and specialist visits							**X**

*t*_1_ baseline, *t*_2_ 3 months, *t*_3_ 6 months, *t*_4_ 12 months

### Participant timeline {13}

### Sample size {14}

In this pilot study, we will recruit 30 patients per arm in order to provide initial insights into the intervention feasibility and protocol as well as preliminary effect size estimates. The aim of this study is not hypothesis testing, and the power level is therefore not a valid consideration for sample size [28, 29]. The sample size for this study (*n* = 60) falls within the range of sample size recommendations for pilot studies of this nature [28, 29].

### Recruitment {15}

Participants are recruited through the hospital cancer outpatient clinics and therapy units. Research nurses and designated health professionals identify potential participants. Potential participants are reviewed by a member of the treating team and asked if they would like to be approached by a research nurse or designated health professionals for consent to participate in the study. Participants are given as much time as possible to consider their participation and are encouraged to take the information away and discuss participation in the trial with family, friends, and their GP if they so wish to. Participants are also encouraged to ask the research nurses, their treating doctors, or nursing staff any questions in relation to their participation.

## Assignment of interventions: allocation

### Sequence generation {16a}

Computer-generated random numbers are used to allocate participants in a 1:1 ratio by a researcher not involved in recruitment, intervention implementation, or data collection. Randomisation is blocked using random permuted blocks of eight and four to ensure that the groups are balanced periodically within stratification groups. Stratification groups include patients who have received (1) surgery only, (2) surgery and radiation only, (3) surgery and chemotherapy ± radiation and are Her2 negative, and (4) surgery and chemotherapy ± radiation and are Her2 positive. These stratification groups were chosen, with clinician input, to allow learnings for patients with different treatment pathways with different follow-up needs to inform the future definitive trial.

### Concealment mechanism {16b}

Allocation sequence is implemented using sequentially numbered opaque, sealed envelopes. Envelopes are only accessed by the research nurse to randomise the patient once recruitment and baseline data has been collected.

### Implementation {16c}

Allocation sequence is generated by a researcher not involved in recruitment or data collection. Patients are enrolled by a research nurse who collects baseline data prior to randomisation. Enrolling nurses assign participants to the intervention after baseline data collection.

## Assignment of interventions: blinding

### Who will be blinded {17a}

After assignment to the intervention, only outcome assessors are blinded to group allocation. Where participants opt to complete their data collection by phone, they are advised not to reveal their group allocation to the outcome assessor. Due to the nature of the intervention, no participants or treating clinicians are blinded.

### Procedure for unblinding if needed {17b}

No unblinding procedures required as only outcome assessors and data analysts are blinded.

## Data collection and management

### Plans for assessment and collection of outcomes {18a}

Patient-reported outcomes are self-administered using online surveys or administered in person or via telephone with an outcome assessor trained in the administration of the study instruments. The description of study instruments is listed in Table 3.

The primary outcome is HRQoL as measured by Functional Assessment of Cancer Therapy—Breast Cancer (FACT-B) [26]. This validated and reliable instrument is well-used in cancer survivors undergoing and beyond treatment [27], and it captures key domains of HRQoL and key symptoms that are relevant to the EMINENT intervention.

The secondary outcomes are listed below:

- Patient experience of care as measured by the Picker Patient Experience 15 (PPE-15) questionnaire [30]. The PPE-15 highlights aspects of care that need improvement to monitor performance and care. It consists of 15 questions distributed to seven dimensions of care: respect, coordination, information/communication/education, physical comfort, emotional support, involvement of relatives, and transitions to community [31].
- Dietary behaviours, specifically usual vegetable intake and usual fruit intake, as measured by two short dietary questions from the National Nutrition Survey [32], which have been validated in the Australian population. Both questions discriminate between groups with significantly different fruit and vegetable intakes. In administering these questions, information about which foods are included as vegetables and fruits is provided and serve sizes are described.
- Physical activity as measured by the Active Australia Survey [33] which is designed to measure participation in leisure-time physical activity, and a single item from the International Physical Activity Questionnaire [34] will be used to measure sedentary behaviours.
- Financial toxicity as measured by the 11-item Comprehensive Score for financial Toxicity (COST)-Functional Assessment of Chronic Illness Therapy (FACIT) tool [35]. This tool is valid and reliable in measuring financial toxicity in patients with cancer [36].
- Adherence to clinical assessments including annual mammography, annual physical examination, and endocrine therapy as measured by hospital records.
- Emergency presentations and hospitalizations as recorded from hospital records.
- Satisfaction of care as measured by a 0–10 numerical analogue scale with 0 being the least satisfied and 10 being the most satisfied, supplemented with short, structured qualitative questions.
- Process outcomes, including completion of intervention components, as measured by completion of intervention materials such as SCPs and checklists, number and length of clinical encounters recorded from MBS data and hospital records, and barriers and facilitators to implementation as explored through semi-structured interviews with patient participants, their nominated carer, breast cancer nurses, GPs, and other healthcare providers including other nurses, and hospital- and community-based rehabilitation providers.
- Health resource utilisation assessing both health service use and participant out-of-pocket costs including MBS and PBS administrative data sets. These data inform participants’ utilisation of services that qualify under the MBS as well as medications dispensed under the PBS. It is planned that the economic evaluation may be reported separately from the main trial.

### Plans to promote participant retention and complete follow-up {18b}

Participants who deviate from the protocol are not withdrawn from the trial. Participants who withdraw from the trial nominate the degree to which they withdraw (i.e. whether they withdraw from active data collection ± passive data collection such as MBS/PBS data).

### Data management {19}

All participant characteristic and outcome data are entered directly into REDCap (Research Electronic Data CAPture – Vanderbilt University, hosted at Queensland University of Technology) by the research nurse ± the participants through self-administered online survey. To ensure data quality, the database is designed with branching logic, data validation, and range checks for data values, where possible.

All source data, clinical records, and laboratory data relating to the study will be archived at the clinical site as appropriate for 15 years after the completion of the study. All data will be available for retrospective review or audit. No study document will be destroyed without prior written agreement between the responsible organisation and the investigator. If the investigator wishes to assign the study records to another party or move them to another location, he/she must notify the responsible organisation in writing of the new responsible person and/or the new location.

### Confidentiality {27}

Data on potential participants is recorded, including reasons for ineligibility or refusal to participate. Participants are only identified by a unique participant study number on the case report forms and other study documents. Other study-related documents (e.g. signed consent form, participant log) are kept in strict confidence by the investigator.

Participants are informed that data is held on file by the responsible organisations and that these data may be viewed by staff including the study project manager and by external auditors on behalf of the responsible organisations and appropriate regulatory authorities (to include reviewing Human Research Ethics Committee (HREC) and the Research Governance Officers). Participants will be identified in publication and conference presentation reports only in aggregated form. All participant data will be held in strict confidence.

### Plans for collection, laboratory evaluation, and storage of biological specimens for genetic or molecular analysis in this trial/future use {33}

Not applicable. There is no collection of biological specimens in the current trial.

## Statistical methods

### Statistical methods for primary and secondary outcomes {20a}

Descriptive statistics will be used to report on feasibility and process-related elements (e.g. recruitment, intervention, retention rates) as well as clinical and resource outcomes. Preliminary effect size estimates for patient and resource use outcomes will be calculated following intention-to-treat principles using generalised linear mixed models. The distribution of the mixed models will be chosen as appropriate for the data, for example, a linear model for scale data or a Poisson for count data. Models will be adjusted for variables used in stratification of the randomisation process. Residuals of all models will be examined for statistical assumptions using descriptive statistics and plots.

### Interim analyses {21b}

Not applicable. No interim analysis is planned.

### Methods for additional analyses (e.g. subgroup analyses) {20b}

All qualitative interviews with participants assigned to the EMINENT intervention are audio-recorded and transcribed verbatim for analysis guided by the Consolidated Framework for Implementation Research [24].

### Methods in analysis to handle protocol non-adherence and any statistical methods to handle missing data {20c}

Preliminary effect size estimates for patient and resource use outcomes will be calculated following intention-to-treat principles using generalised linear mixed models. Patterns of missing data will be examined using chi-square and *t* tests. Missing data for the outcomes will be accounted for by using mixed models allowing the use of each available case by computing maximum likelihood estimates.

### Plans to give access to the full protocol, participant level-data, and statistical code {31c}

Not applicable. There are no plans for granting public access of the full protocol, participant-level dataset, or statistical code.

## Oversight and monitoring

### Composition of the coordinating centre and trial steering committee {5d}

The Chief investigators are the trial steering committee that will provide all governance to the conduct of the study.

### Composition of the data monitoring committee, its role, and reporting structure {21a}

Not applicable. There is no data monitoring committee established for this pilot trial.

### Adverse event reporting and harms {22}

An adverse event (AE) is any event, side effect, or other untoward medical occurrences that occur in conjunction with the use of the study intervention in humans, whether or not considered to have a causal relationship to the interventions. An AE can, therefore, be any unfavourable and unintended sign (that could include a clinically significant abnormal laboratory finding), symptom, or disease temporally associated with the use of the study intervention, whether or not considered related to the intervention. Conditions recognised as being excluded from AE reporting are as follows: any event, side effect, or other medical occurrences that are anticipated because of the normal course of treatment (standard care). There are no known side effects/adverse events associated with the proposed model of care intervention [9]. Due to the nature of this intervention, there will be no reporting of AE.

### Frequency and plans for auditing trial conduct {23}

There are no plans for auditing trial conduct beyond the independent research governance requirements and annual reporting to the HREC.

### Plans for communicating important protocol amendments to relevant parties (e.g. trial participants, ethical committees) {25}

All agreed protocol amendments are clearly recorded on a protocol amendment form and are signed and dated by the original protocol approving signatories. All protocol amendments will be submitted to the institutional HREC for approval before implementation. The only exception will be when the amendment is necessary to eliminate an immediate hazard to the trial participants. In this case, the necessary action will be taken first, with the relevant protocol amendment following shortly thereafter. Once HREC approval has been granted, investigators and the ANZCTR will be updated.

### Dissemination plans {31a}

It is intended that the findings from this trial will be disseminated at academic and professional conferences and via a manuscript submission to a peer-reviewed journal. Participants will be identified in such reports only in aggregate or by study identification number, gender, and age. There are no publication restrictions.

## Discussion

Despite the strong case for a shared, follow-up care model for breast cancer survivors involving cancer specialists and GPs, barriers to shared care mean that it is not routinely implemented across Australia. These include the need for coordination across multiple providers, the need for improved patient and provider knowledge about the benefits of shared care and how to implement it, insufficient or delayed communication between cancer specialists and GPs, and lack of awareness of available support such as funding models, tools, and resources [11, 12]. The current study aims to address these barriers using a Specialist Cancer Nurse to advise stakeholders of the benefits of shared care (patient and GPs), facilitate effective and timely care coordination, and act as the conduit between the specialist cancer multidisciplinary team and the GPs.

Practical issues for this trial include estimating the time required to coordinate the trial across multiple providers including engaging GPs and fidelity with the intervention components. The proposed study will provide important information on the feasibility of a definitive phase 3 trial for implementing a nurse-enabled, integrated, shared-care model involving cancer specialists and GPs for early breast cancer post-treatment follow-up. The information collected through the trial, qualitative interviews, and economic evaluations are crucial in guiding the development of such a trial.

## Trial status

The protocol published here is version 2.0 dated 24 March 2020. The trial began recruitment on 3 December 2019 and is expected to continue until 20 November 2021.

Trial registration: Australia and New Zealand Clinical Trials Registry, ACTRN12619001594112. Registered on 19 November 2019, https://www.anzctr.org.au/Trial/Registration/TrialReview.aspx?id=378690&isReview=true.

## Supplementary information

**Additional file 1.** Interview Guide for the Semi-Structured Interviews with Patients/Family Members and Health Professionals.

## Data Availability

There are no limitations on investigator access to the trial dataset. The datasets generated and/or analysed during the current study are not going to be made publicly available but will be made available from the corresponding author on reasonable request.

## Abbreviations

HRQoL: Health-related quality of life
GPs: General practitioners
DCIS: Ductal carcinoma in situ
REDCap: Research Electronic Data Capture
ECOG: Eastern Cooperative Oncology Group
PBS: Pharmaceutical Benefits Scheme
MBS: Medicare Benefits Schedule
SCP: Survivorship Care Plan
FACT-B: Functional Assessment of Cancer Therapy—Breast Cancer
PPE-15: Patient Experience of Care
AAS: Active Australia Survey
IPAQ: International Physical Activity Survey
COST-FACIT: Comprehensive Score for Financial Toxicity-Functional Assessment of Chronic Illness Therapy
SACQ: Self-administered Comorbidity Questionnaire
CCI: Charleston Comorbidity Index
HREC: Human Research Ethics Committee
