# Supplementary material for: Implementing a nurse-enabled, integrated, shared-care model involving specialists and general practitioners in breast cancer post-treatment follow-up: a study protocol for a phase II randomised controlled trial (the EMINENT trial)
Source: Trials. 2020 Oct 15;21:855. doi: 10.1186/s13063-020-04740-1 (PMC7558254; doi:10.1186/s13063-020-04740-1)
Supplement: Supplementary file 1 — Additional file 1. Interview Guide for the Semi-Structured Interviews with Patients/Family Members and Health Professionals. [file 13063_2020_4740_MOESM1_ESM.docx]

***Supplementary Material 1. Interview Guide for the Semi-Structured Interviews with Patients/Family Members and Health Professionals***

**EMINENT Trial Interview Guide – Patients/Family Members**

Describe your experience participating in the EMINENT trial.

How did the trial meet your expectations?

What aspects of the trial were valuable to you?

What aspects of the trial were valuable to others (e.g., GPs, other HPs)?

Describe any aspects of the trial that were challenging for you

**EMINENT Trial Interview Guide – Health Professionals**

Describe your experience participating in the EMINENT trial.

How did the trial meet your expectations?

What aspects of the trial were valuable to you?

What aspects of the trial were valuable to patients/carers?

Describe any aspects of the trial that you thought were challenging

Describe any aspects of the intervention that you thought were challenging for patients/carers

How does the intervention compare to other alternatives that may have been considered or that you know about?

What kinds of changes or alterations do you think you will need to make to the intervention so it will work effectively in your setting?

Are there components that should not be altered?

What is your perception of the quality of the supporting materials, packaging, and bundling of the intervention for implementation?

What costs were incurred to implement the intervention?
